# Ameliorative effect of *Ruellia tuberosa* L. on hyperglycemia in type 2 diabetes mellitus and glucose uptake in mouse C2C12 myoblasts

**DOI:** 10.1002/fsn3.840

**Published:** 2018-10-10

**Authors:** Chih‐Yuan Ko, Ru‐Hai Lin, Yi‐Ming Zeng, Wen‐Chang Chang, Da‐Wei Huang, James Swi‐Bea Wu, Yu‐Fang Chang, Szu‐Chuan Shen

**Affiliations:** ^1^ Department of Respiratory and Critical Care Medicine the Second Affiliated Hospital of Fujian Medical University Quanzhou China; ^2^ Respiratory Medicine Center of Fujian Province Quanzhou China; ^3^ Key Laboratory of Fujian Medical University Fujian Province University Quanzhou China; ^4^ Department of Endocrinology and Metabolism the Second Affiliated Hospital of Fujian Medical University Quanzhou China; ^5^ Department of Food Science National Chiayi University Chiayi City Taiwan; ^6^ Department of Biotechnology Southern Taiwan University of Science and Technology Tainan City Taiwan; ^7^ Graduate Institute of Food Science and Technology National Taiwan University Taipei Taiwan; ^8^ Department of Human Development and Family Studies National Taiwan Normal University Taipei Taiwan; ^9^ Grauduate Program of Nutrition Science National Taiwan Normal University Taipei Taiwan

**Keywords:** C2C12 cells, hyperglycemia, insulin resistance, *Ruellia tuberosa* L., type 2 diabetes mellitus

## Abstract

*Ruellia tuberosa* L. (RTL) exhibits a wide range of phytochemical activities, for example, on treatment of diabetes mellitus (DM), in Orient. There is, however, few study regarding the effect of RTL on glycemic‐related homeostasis in type 2 DM (T2DM). We investigated the effect of RTL aqueous and ethanolic extracts on hypoglycemia in high‐fat diet (HFD)‐fed plus streptozotocin (STZ)‐induced T2DM rats, and examined the effect of RTL on glucose uptake in tumor necrosis factor‐α‐induced insulin‐resistant mouse C2C12 myoblasts, a mouse skeletal muscle cell line. The administration of 100 or 400 mg kg BW^−1^ day^−1^ of RTL aqueous or ethanolic extracts once a day for 4 weeks significantly ameliorated hyperglycemia, hyperinsulinemia, and the insulin resistance (IR) index in diabetic rats. RTL either aqueous or ethanolic extract at a concentration of 25–800 μg/ml significantly improved glucose uptake in insulin‐resistant mouse C2C12 myoblasts, indicating inhibiting the IR in skeletal muscles. These evidences suggest that RTL ameliorates hyperglycemia in HFD/STZ‐induced T2DM rats may be attributed to the alleviation of IR in skeletal muscles.

## 1 INTRODUCTION

Type 2 diabetes mellitus (T2DM) occurs mainly due to insufficient or a lack of insulin secretion from the pancreatic β cells and insulin insensitivity for a tissue, resulting in insulin cannot effectively be exerted, causing insulin resistance (IR; Boden et al., 2005; Tuncman et al., 2006). To maintain homeostasis of blood glucose in the body, pancreatic β cells compensatorily secrete more insulin to elevate the blood insulin level, subsequently contributing to hyperinsulinemia (ADA, 2015; Jiang, Chang, Tai, Chen, & Chuang, 2012; Samuel & Shulman, 2012).

Various factors are involved in the pathogenesis of IR, such as ectopic lipid metabolite accumulation, unfolded protein response activation, and innate immune responses (Samuel & Shulman, 2012). These pathogenic mechanisms may be related to abnormal fat metabolism and synthesis or energy consumption caused by the accumulation of ectopic lipid metabolites (Chang, Shen, & Wu, 2013; Chang et al., 2015; Longato, Tong, Wands, & de la Monte, 2012; McNeilly, Williamson, Sutherland, Balfour, & Stewart, 2011). It may eventually generate some specific lipid metabolites that converge to the liver and skeletal muscle, resulting in the destruction of the insulin signaling pathway and the occurrence of IR (Chang & Shen, 2013; Chang et al., 2013, 2015). Previous studies reported that tumor necrosis factor‐α (TNF‐α) may activate the inhibitor of nuclear factor κ‐B kinase and c‐Jun N‐terminal protein kinase (JNK) pathways, resulting in IR in skeletal muscle cells (Boden et al., 2005; Samuel & Shulman, 2012; Tuncman et al., 2006).

Evidence has shown that *Ruellia tuberosa* L. (RTL) possesses antioxidant, anti‐inflammation, anticancer, and other physiological capabilities (Chothani, Patel, Mishra, & Vaghasiya, 2010). RTL can also regulate the blood glucose and lipid balance in alloxan‐induced diabetic rats and rabbits (Ananthakrishnan & Doss, 2010, 2013; Rajan, Kishor Kumar, Satheesh Kumar, Swathi, & Haritha, 2012; Shahwara et al., 2011; Wulan, Utomo, & Mahdi, 2015). However, there has been no reported literature regarding the effect of RTL on blood glucose homeostasis in T2DM. The objectives of this study were to investigate the ameliorative effect of RTL on hyperglycemia in in vivo high‐fat diet (HFD)‐fed streptozotocin (STZ)‐induced diabetic rats, and glucose uptake in in vitro TNF‐α‐induced insulin‐resistant mouse C2C12 myoblasts.

## 2 METHODS AND MATERIALS

### 2.1 Preparation of crude RTL extracts

The stems and leaves of RTL were collected from the Herb Light farm, Yi‐Lan County, Taiwan, in May of 2014. RTL were washed, drained, weighed, sliced, and freeze‐dried. Each 1 g of dried material was extracted with 6 ml of distilled water and 95% ethanol (1:6, w/v) at 4°C for 72 hr, and filtered through cheesecloth. The filtrate is filtered twice through Whatman No. 1 filter paper, and then, centrifuged at 4,700 *g* for 20 min. The supernatant is vacuum concentrated using a rotary evaporator below 40°C. The concentrate was freeze‐dried into a powder and stored at 80°C until used. The extraction rate of RTL water extracts (RTLW) and RTL ethanolic extracts (RTLE) was 11.41% and 2.01%, respectively. The appearance of both crude extract powders was brownish green after being freeze‐dried.

### 2.2 Animal experimental procedure

Male Wistar rats (4‐weeks‐old) were procured from the National Laboratory Animal Center, Taipei, Taiwan. The room conditions and treatment procedures were in accordance with the National Institutes of Health Guide for the Care and Use of Laboratory Animals, and all of the protocols were approved by the Institutional Animal Care and Use Committee of National Taiwan Normal University, Taipei, Taiwan (proved No. 103042). The rats were maintained in a temperature‐ (22 ± 1°C) and humidity‐controlled (50% ± 20%) room under a 12‐h light/dark cycle (lights on from 08:00 to 20:00) with free access to food and water. After 1‐week adaptation, the rats were fed with HFD (60% calories from fat) for 4 weeks. In the 5th and 6th weeks, the STZ (28 and 15 mg/kg body weight, respectively, is dissolved in 0.1 M sodium citrate buffer at pH 4.5) is intraperitoneally injected into each HFD rat to induce diabetes. After the STZ injection, rats were supplied with drinking water containing 5% sucrose for 48 hrs, in order to reduce early death due to insulin discharge from partially injured pancreatic islets. Seventy‐two hours later, rats were checked for hyperglycemia. For the animal experimental design, the rats were divided into seven groups (each contains six rats): Group 1 consists of rats fed a normal diet for 11 weeks; Group 2 consists of diabetic rats fed an HFD (60% calories from fat) for 11 weeks as the negative control; Group 3 consists of diabetic rats fed an HFD for 11 weeks and orally administered pioglitazone (Pio; 30 mg/kg body weight) daily during the last 4 weeks (7th week–11th week) as the positive control; Groups 4 and 5 consisted of diabetic rats fed an HFD and orally administered RTLW (100 or 400 mg/kg body weight, respectively) daily during the last 4 weeks (7th week–11th week); Groups 6 and 7 consisted of diabetic rats fed an HFD and orally administered RTLE (100 or 400 mg/kg body weight, respectively) daily during the last 4 weeks (7th week–11th week). All rats were sacrificed at the end of the experiment, blood samples were collected, and the biochemical analysis was conducted. The livers were stored at −80°C for Western blot analysis.

### 2.3 Blood sample preparation

Blood samples were collected and allowed to clot for 30 min at room temperature, centrifuged at 3,000 *g* for 10 min twice to obtain the serum, which is stored at −80°C till it was used.

### 2.4 Serum biochemical measurements

Glucose concentration of serum was determined using a glucose enzymatic kit (Crumlin Co., Antrim, UK). The concentration of insulin was measured by insulin kit (Crumlin Co.).

### 2.5 Homeostasis model assessment of insulin resistance

The homeostasis model assessment of insulin resistance (HOMA‐IR) index was calculated as (fasting insulin μU/L) × (fasting glucose mmol/L)/22.5 (Matthews et al., 1985).

### 2.6 Quantitative insulin sensitivity check index

The quantitative insulin sensitivity check index (QUICKI) was calculated using the following equation: 1/(log [fasting glucose (mg/dl)] + log [fasting insulin (μU/ml)]) (Katz et al., 2000; Mari, Ahrén, & Pacini, 2005).

### 2.7 Cell culture

The experiments were performed on mouse C2C12 myoblasts; a mouse skeletal muscle cell line derived from a myotube cell (around 6 days). The cells were incubated in DMEM containing 10% FBS (Invitrogen Corporation, Camarillo, CA, USA) in 10 cm Petri dishes at 37°C and 5% CO_2_. Experiments were performed on cells that were 80%–90% confluent.

### 2.8 TNF‐α induction of IR in C2C12 cells

The induction of IR in cell line referred to the method reported by previous study with minor modifications (Chang & Shen, 2013). C2C12 cells were seeded in 10 cm dishes and incubated at 37°C for 48 hr to 80% confluence. Serum‐free DMEM medium containing different concentrations (0, 2.5, 5, 10, 20, 40 ng/ml) recombinant mouse TNF‐α was then added and incubated for 2 hr to induce IR.

### 2.9 Uptake of fluorescent 2‐[*N*‐(7‐nitrobenz‐2‐oxa‐1,3‐diazol‐4‐yl) amino]‐2‐deoxy‐d‐glucose in C2C12 cells

The C2C12 cells were seeded in 10 cm dishes and then incubated at 37°C for 48 hr to achieve 80% confluence. Serum‐free DMEM medium containing 10 ng/ml recombinant mouse TNF‐α was added before incubating for 2 hr to induce IR. The cells were then transferred to another DMEM medium containing 5 mM glucose, without (basal) or with 100 nM insulin and 0, 25, 50, 100, 200, 400, 800 μg/ml RTLW/RTLE and incubated for 30 min at 37°C. An assay of glucose uptake was then performed as described previously (Chang et al., 2015). The fluorescence intensity of the cell suspension was evaluated using flow cytometry (FACScan; Becton Dickinson, Bellport, NY, USA) at an excitation wavelength of 488 nm and an emission wavelength of 542 nm. Fluorescence intensity reflected the cellular uptake of 2‐[*N*‐(7‐nitrobenz‐2‐oxa‐1,3‐diazol‐4‐yl) amino]‐2‐deoxy‐d‐glucose (2‐NBDG), was calculated as Amelioration rate (%) = [(fluorescence intensity of phenolic acid − treated group) − (fluorescence intensity of TNF‐α − treated group)]/(fluorescence intensity of TNF‐α − treated group) × 100%.

### 2.10 Statistical analysis

Values are presented as the mean ± standard deviation (*SD*), which is analyzed statistically with SAS Version 9.4 (SAS Institute Inc, Cary, NC, USA) using one‐way ANOVA and Duncan's new multiple range tests. *p* < 0.05 is considered statistically significant.

## 3 RESULTS

### 3.1 Effect of RTL on body weight, dietary, water, and caloric intakes in HFD‐fed STZ‐induced diabetic rats

**Figure 1:**
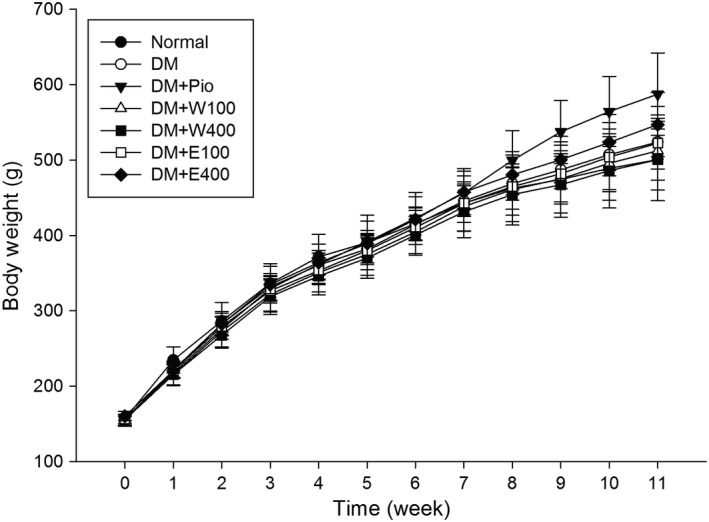
The changes of body weight of high‐fat diet plus streptozotocin (STZ)‐induced type 2 diabetic rats treated with *Ruellia tuberosa* L. (RTL) extracts. Normal: Normal diet from 0 week to 11th week; DM: High‐fat diet (HFD; 60% fat) plus STZ (28 mg/kg body weight, i.p.) from 0 week to 11th week; DM + Pio: DM rats gavaged Pioglitazone (30 mg/kg body weight) from 7th week to 11th week; DM + W100: DM rats gavaged RTL water extract (100 mg/kg body weight) from 7th week to 11th week; DM + W400: DM rats gavaged RTL water extract (400 mg/kg body weight) from 7th week to 11th week; DM + E100: DM rats gavaged RTL ethanol extract (100 mg/kg body weight) from 7th week to 11th week; DM + E400: DM rats gavaged RTL ethanol extract (400 mg/kg body weight) from 7th week to 11th week. Values were calculated as mean ± *SD*,* n* = 6 for each group

As shown in Figure 1, the body weight of rats increased regularly as time went by. Rats received RTL treatment at 7th week. The body weight of DM + Pio group increased faster and the average body weight at 11th week was 587.4 ± 54.7 g, significantly higher than those of the other groups (*p* < 0.05).

Table 1 shows that the average water intake of the normal group (75.1 ± 1.5 ml rat^−1^ day^−1^) was significantly higher than that of the DM + Pio and DM + W400 groups (55 ml rat^−1^ day^−1^; *p* < 0.05). The dietary intake (30.3 ± 0.5 g rat^−1^ day^−1^) of the normal group was significantly higher than those of the other groups (*p* < 0.05). The dietary intake of the DM + Pio group was 21.6 ± 0.3 g rat^−1^ day^−1^ (*p* < 0.05), which was significantly higher than that of the DM group, and there was no significant difference among the other groups. The caloric intake of the normal group (141.5 ± 2.3 kcal rat^−1^ day^−1^) was the lowest compared with the other groups. Furthermore, the caloric intake of the DM + Pio group (180.2 ± 2.1 kcal rat^−1^ day^−1^) was higher than those of the DM, DM + W100, and DM + W400 groups (*p* < 0.05).

**Table 1:** Diet intake, drink intake, and calorie intake in high‐fat diet plus streptozotocin‐induced type 2 diabetic rats after treated with *Ruellia tuberosa* L. extracts for 4 weeks

	Normal	DM	DM + Pio	DM + W100	DM + W400	DM + E100	DM + E400
Drink intake (ml rat^−1^ day^−1^)	75.1 ± 1.5^a^	61.2 ± 0.8^ab^	55.3 ± 1.0^b^	63.3 ± 1.3^ab^	55.1 ± 1.1^b^	65.8 ± 1.4^ab^	63.1 ± 1.2^ab^
Diet intake (g rat^−1^ day^−1^)	30.3 ± 0.5^a^	21.6 ± 0.3^c^	25.7 ± 0.3^b^	21.3 ± 0.2^c^	21.1 ± 0.3^c^	22.3 ± 0.3^bc^	24.2 ± 0.3^bc^
Calorie intake (kcal rat^−1^ day^−1^)	141.5 ± 2.3^ab^	151.1 ± 2.2^b^	180.2 ± 2.1^a^	149.3 ± 1.6^b^	147.9 ± 1.8^b^	156.2 ± 2.1^ab^	169.3 ± 2.3^ab^

Note

Abbreviations are as in Figure 1. Values were calculated as mean ± *SD*,* n* = 6 for each group. a–c letter is significantly different among all sample tested (*p* < 0.05).

### 3.2 Effect of RTL on fasting serum glucose and insulin levels in HFD‐fed STZ‐treated rats

The fasting serum glucose of HFD‐fed STZ‐induced rats was (253.9 ± 97.1 mg/dl) and higher than that of the normal group (93.6 ± 9.5 mg/dl) at 7th week, indicating the successful induction of diabetes (data not shown).

**Figure 2:**
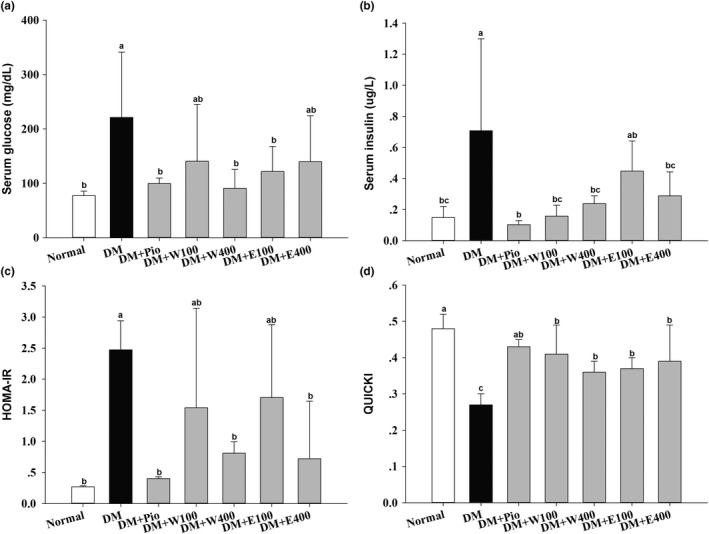
Serum glucose (a), serum insulin (b), homeostasis model assessment‐insulin resistance (HOMA‐IR) index (c), and quantitative insulin sensitivity check index (QUICKI) (d) of high‐fat diet plus streptozotocin (STZ)‐induced type 2 diabetic rats treated with *Ruellia tuberosa* L. (RTL) extracts for 4 weeks. Abbreviations are as in Figure 1. Values were calculated as mean ± *SD*,* n* = 6 for each group. a–c letter is significantly different among all sample tested (*p* < 0.05)

After 4 weeks of treatment (at 11th week), the serum glucose level of the DM group (221.3 ± 119.9 mg/dl), shown in Figure 2a, was significantly higher than that of the normal group (77.7 ± 8.2 mg/dl; *p* < 0.05). Meanwhile, after 4 weeks of treatment, the serum glucose levels of the DM + Pio, DM + W400, and DM + E100 groups were 99.7 ± 10.0, 90.8 ± 34.7, and 121.8 ± 45.9 mg/dl, respectively, which were significantly lower than that of the DM group (*p* < 0.05). The serum insulin level of the DM group (0.71 ± 0.59 μg/L), shown in Figure 2b, was significantly increased compared with that of the normal group (0.15 ± 0.07 μg/L; *p* < 0.05). After 4 weeks of treatment, the serum insulin levels of the DM + Pio, DM + W100, DM + W400, and DM + E400 groups were 0.10 ± 0.03, 0.16 ± 0.07, 0.24 ± 0.05, and 0.29 ± 0.15 μg/L, respectively, which were significantly lower than that of the DM group (*p* < 0.05). The above observation from fasting serum glucose and insulin levels also suggests the successful induction of T2DM in rats.

### 3.3 Effect of RTL on IR in HFD‐fed STZ‐induced T2DM rats

The HOMA‐IR of the DM group was 2.47 ± 0.47, which was significantly higher than that of the normal group (0.27 ± 0.02, *p* < 0.05). The HOMA‐IR of the DM + Pio, DM + W400, and DM + E400 groups was 0.40 ± 0.03, 0.81 ± 0.18, and 0.72 ± 0.92, respectively, which were significantly lower than that of the DM group (*p* < 0.05; Figure 2c). Figure 2d shows that the QUICKI of the normal group was 0.48 ± 0.04, which was significantly higher than those of the other groups (*p* < 0.05). The QUICKI of the DM group was the lowest among all the groups (0.27 ± 0.03, *p* < 0.05).

### 3.4 Effect of RTL on survival rate in mouse C2C12 myoblasts

Concentrations of either RTLW or RTLE (25, 50, 100, 200, 400, and 800 μg/ml), coculture with C2C12 cells for 24 hr, exhibited that cell survival rates were above 80% without inhibition of cell growth phenomenon (Table 2).

**Table 2:** Concentration effect of *Ruellia tuberosa* L. (RTL; containing stems and leaves) water and ethanol extracts on the cell viability of mouse C2C12 myoblasts

	Cell viability (%)^a^					
	RTL extracts (μg/ml)					
	25	50	100	200	400	800
Water extracts	100.5 ± 9.2	97.8 ± 4.6	97.6 ± 1.4	90.1 ± 4.7	91.7 ± 2.9	91.9 ± 3.3
Ethanol extracts	99.6 ± 4.4	105.5 ± 6.6	98.0 ± 4.9	86.2 ± 5.6	102.3 ± 12.8	108.1 ± 11.4

Notes

Each value is means ± *SD* (*n* = 3).

$$\text{Cell viability}\;\left(\%\right)=\frac{\text{Sample group}-\text{Blank group}}{\text{Control group}-\text{Blank group}}\times 100\%.$$

a Values are percentage relative to control value (100%).

### 3.5 Effect of RTL on glucose uptake in mouse C2C12 myoblasts

**Figure 3:**
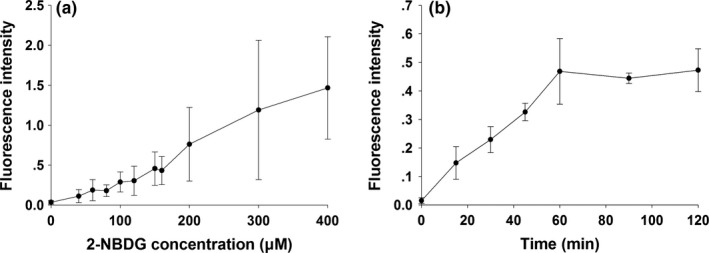
Changes of fluorescence intensity in mouse C2C12 myoblasts incubated with various concentrations of 2‐NBDG (a) and different response times (b)

The glucose intake in the C2C12 cells showed a dose‐dependent manner. The fluorescence increasing as the concentration increased, the amount of fluorescence per unit of protein in different 2‐NBDG concentration (0, 40, 60, 80, 100, 120, 160, 200, 300, 400 μM) in order of 0.04 ± 0.02, 0.11 ± 0.08, 0.19 ± 0.13, 0.18 ± 0.07, 0.29 ± 0.13, 0.30 ± 0.18, 0.43 ± 0.18, 0.76 ± 0.46, 1.19 ± 0.87, and 1.47 ± 0.64 were measured, respectively (Figure 3a). The glucose uptake by C2C12 cells also increased with the higher concentration. The higher the fluorescence intensity was measured at different action times of 15, 30, 45, 60, 90, and 120 min. The amount of fluorescence in cellular proteins was in the order of 0.02 ± 0.01, 0.15 ± 0.06, 0.23 ± 0.05, 0.33 ± 0.03, 0.47 ± 0.11, 0.44 ± 0.02, and 0.47 ± 0.08, respectively (Figure 3b).

**Figure 4:**
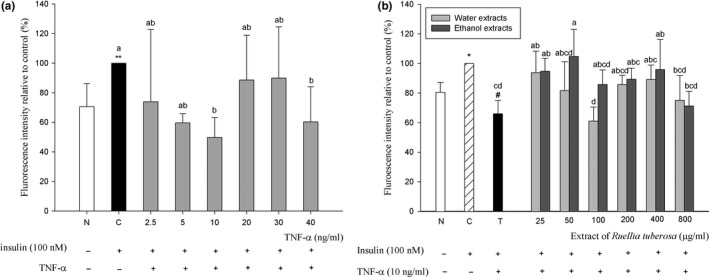
Inhibition of TNF‐α on glucose uptake in mouse C2C12 myoblasts (a), and effect of extract from *Ruellia tuberosa* L. (RTL) on glucose uptake in TNF‐α‐induced insulin‐resistant mouse C2C12 myoblasts (b). C: Cells incubated with DMEM medium containing 100 nM insulin; N: Cells incubated with DMEM medium; T: Cells induced insulin resistance by TNF‐α. *p < 0.05, *^*^ *p* < 0.01: Significantly different from N. a–b letter is significantly different from C (*p* < 0.05). a–d letter is significantly different from T (*p* < 0.05)

The addition of TNF‐α reduced the effect of insulin on glucose uptake in C2C12 cells compared with the control group without TNF‐α. At 10 and 40 ng/ml, RTL extracts were lower cell glucose uptake effect better than that of the control group, respectively, can reduce 50.2% and 39.7% (*p* < 0.05; Figure 4a).

Concentrations of 25, 50, 200, 400, and 800 μg/ml of RTLW can improve glucose uptake of IR in C2C12 cells. The best effect of concentration was at 25 μg/ml (*p* < 0.05), and the improvement rate was 27.8%. Concentrations of 25, 50, 100, 200, 400, and 800 μg/ml of RTLE can improve glucose uptake of IR in C2C12 cells. The efficiency levels were 25, 50, and 400 μg/ml (*p* < 0.05), and the improvement rates were 28.7%, 38.8%, and 29.9%, respectively (Figure 4b).

## 4 DISCUSSION

Evidence indicates that RTL has various bioactivities, such as antioxidant, antimicrobial, anticancer, and anti‐inflammatory activities, and it also reduces thirst (Chothani et al., 2010). However, currently, RTL‐related DM studies using alloxan‐induced models are predominant (Ananthakrishnan & Doss, 2010, 2013; Shahwara et al., 2011), and a few surveys have explored the effect of RTL on the HFD‐fed STZ‐induced T2DM paradigm and the IR mechanism via inflammation, causing TNF‐α activation in skeletal muscle cells. Herein, this study employed a diabetic rodent model to investigate both RTLW and RTLE for the improvement of IR, and to verify the glucose uptake ability in mouse C2C12 myoblasts.

Our animal study results indicate that the administration of 100 and 400 mg kg BW^−1^ day^−1^ of RTL aqueous or ethanolic extract once a day for 4 weeks does not influence their body weight, water consumption, and caloric intake. The dietary intake of the normal group was significantly higher than those of the other groups; however, the caloric intake of the normal group was significantly lower than those of the other groups. Because the normal group consumed a standard laboratory chow diet under normal circumstances, overfeeding does not occur, which does not lead to excessive caloric intake. In contrast, the mean daily caloric intake was significantly higher in the DM + Pio group, which may be one of the reasons why the average body weight of the DM + Pio group was significantly higher than those of the other groups after 4‐week administration of RTL extracts. Furthermore, pioglitazone belongs to the thiazolidinedione group of hypoglycemic drugs that promote adipocyte fatty acid intake, fat synthesis, and differentiation through the activation of peroxisome proliferator‐activated receptor‐γ (Kersten, Desvergne, & Wahli, 2000; Vamecq & Latruffe, 1999). Research shows that pioglitazone can increase the total body water of diabetic patients, causing edema (Hollenberg, 2003), suggesting that the significantly elevated body weight of the DM + Pio group is caused by pioglitazone.

There is no doubt that HFD with STZ intraperitoneal injection successfully induces T2DM in rats (Sasidharan et al., 2013). Our previous study showed that the administration of methylglyoxal (MG) after 8 weeks significantly elevates fasting blood sugar. MG is one of the metabolites in glycolysis in which its level is higher in patients with DM than in the general population (Chang et al., 2013). Hence, we propose that the higher fasting blood sugar in DM group may be caused by abnormal carbohydrate metabolism. Excess fat in an HFD causes IR that leads to compensatory insulin secretion from the pancreas and results in hyperinsulinemia in rats (Longato et al., 2012; McNeilly et al., 2011). In the present study, HFD‐fed STZ‐induced diabetic rats exhibited both hyperglycemia and hyperinsulinemia, indicating the successful induction of T2DM. However, both RTLW and RTLE significantly ameliorated serum glucose and insulin levels, suggesting hypoglycemic and hypoinsulinemic abilities in T2DM rats. RTL extracts also improve the oral glucose tolerance test in HFD‐fed STZ‐treated rats, and the findings have demonstrated in our previous study (Chang et al., 2018). HOMA‐IR and QUICKI are indexes that used clinically to assess IR in patients with DM. HOMA‐IR values >2.0 or QUICKI values <0.339 indicate IR in humans (Katz et al., 2000; Mari et al., 2005; Matthews et al., 1985). Our results indicate that the administration of RTL extracts significantly decreases HOMA‐IR values and increases QUICKI values, indicating that RTL alleviates IR in diabetic rats. A previous study has reported that feeding RTLE to diabetic rats exhibits benefit in regulating blood lipids (Ananthakrishnan & Doss, 2013). Apart from dyslipidemia, the most typical symptoms of DM are hyperglycemia and hyperinsulinemia. Wulan et al. (2015) have shown functions of RTL on inhibiting the activity of α‐amylase, delaying carbohydrate decomposition, and reducing postprandial blood glucose disproportion in diabetes. Various phytochemicals of RTL components were reported, including betulin, vanillic acid, flavonoids, apigenin, luteolin, or flavone glycoside (Lin, Huang, Cheng, Sheu, & Chen, 2006; Nair & Subramanian, 1974; Wagner, Danninger, Iyengar, Seligmann, & Farkas, 1971), among which betulin has a major role, act as noncompetitive inhibitors of α‐amylase to achieve hypoglycemia (Wulan et al., 2015). However, isolating and identifying the active components from RTL extracts through column chromatography, high‐performance liquid chromatography, and confirming their benefit functions are currently on the way in our laboratory.

Our data reveal that RTL extracts can ameliorate IR and glucose tolerance in HFD‐fed STZ‐induced T2DM rats. The mechanism of IR in skeletal muscle cells was caused by an HFD paradigm (Samuel & Shulman, 2012). However, in cell experiments, RTL was extracted using different solvents and the resulting extract was found to have caused cancer cell toxicity (Lin et al., 2006). Some studies obtained different layers of extraction using different solvents to extract RTL, which was found to have free radicals (DPPH, hydrogen peroxide, and ROO¯) scavenging activity, and one of the best effects was obtained with extraction with ethyl acetate (Chen, Wu, Shieh, Kuo, & Hsieh, 2006; Phakeovilay et al., 2013). However, currently, the optimal concentration of RTL extract to be used has not been determined. Higher concentrations of RTL extracts may cause cytotoxicity; therefore, we referred to our previous study on the application of plant extract to cells in order to select an effective concentration (Chang et al., 2013). In this study, we slightly modified the testing concentration (25–800 μg/ml) to examine cell viability and obtained 800 μg/ml of water and ethanolic extracts without toxicity for mouse C2C12 blasts, a mouse skeletal mouse cell line. Concentrations of 25, 50, 100, 200, 400, and 800 μg/ml of RTL extracts were used for studying subsequent glucose uptake in C2C12 cells induced by TNF‐α. When the concentration of 2‐NBDG >200 μM, glucose uptake by cells becomes less stable, resulting in a large change in the fluorescence value. This may be due to an increase in the collision between molecules and the gradual loss of energy in molecules as the 2‐NBDG concentration increases, leading to a self‐quenching situation (Chang et al., 2013), rendering the measured fluorescence value less stable. The measured fluorescence begins to reach saturation when the reaction continues for more than an hour. This phenomenon may be because C2C12 cells uptake of glucose has been saturated, leading to cellular uptake of fluorescent glucose 2‐NBDG has been metabolically utilized by cells to reduce the amount of fluorescence. Finally, in accordance with previous studies (Arora & Dey, 2014; Yang et al., 2013), follow‐up experiments were performed with 100 μM of 2‐NBDG for 30 min.

To investigate the effect of both RTLW and RTLE on glucose uptake in C2C12 cells, the concentrations of 25–800 μg/ml of RTL extracts were used to test glucose uptake induced by TNF‐α in an IR cell model. Del Aguila, Claffey, and Kirwan (1999) utilized C2C12 cells induced by TNF‐α at a concentration of 10 ng/ml for 1 hr and found that under these conditions, the activity of IRS‐1/2 is inhibited, resulting in a decrease in PI3‐kinase activity and The present study demonstrates inhibition of P42^MARK^ and P44^MARK^ phosphorylation, thereby enabling insulin to stimulate the decrease in glucose uptake of C2C12 cells. When TNF‐α binds to its receptor, it activates the JNK pathway and directly inhibits the activity of IRS‐1/2, resulting in the failure of the insulin signaling pathway, leading to cellular IR emergence (Boden et al., 2005; Samuel & Shulman, 2012; Tuncman et al., 2006). Thus, in the present study, a concentration of 10 ng/ml of TNF‐α was also used as a cellular model for the induction of IR in C2C12 cells. We demonstrate that various concentrations of both RTLW and RTLE enhanced glucose uptake ability in TNF‐α‐induced insulin‐resistant C2C12 cells model. It is presumed that the effect of RTL on improving the IR of C2C12 may be due to RTL contains abundant phytochemicals that possess antioxidative ability (Chen et al., 2006; Phakeovilay et al., 2013), anti‐inflammatory activity (Alam et al., 2009), etc. Our data reveal that RTL extracts may improve IR in animal or cell experiments. Our results also show that alleviating skeletal muscle IR may play an important role on ameliorating hyperglycemia and hyperinsulinemia in DM.

## 5 CONCLUSIONS

The present study demonstrates that both RTL extracts may ameliorate hyperglycemia and IR index in HFD‐fed STZ‐induced T2DM rats. The result from cell test also indicates that both RTLW and RTLE may increase glucose uptake in TNF‐α‐induced insulin‐resistant mouse C2C12 myoblasts, indicating the decrease in IR in mouse skeletal muscles. These findings suggest that RTL alleviates hyperglycemia may be mainly/partially attributed to the suppression of IR in skeletal muscles, and subsequently prevent the progression of T2DM.

## ETHICAL STATEMENT

The study was performed in accordance with the ethical guidelines of the Institutional Animal Care and Use Committee of National Taiwan Normal University, Taipei, Taiwan (proved No. 103042).

## CONFLICT OF INTEREST

The authors declare that they do not have any conflict of interest.
