# Supplementary material for: Ameliorative effect of Ruellia tuberosa L. on hyperglycemia in type 2 diabetes mellitus and glucose uptake in mouse C2C12 myoblasts
Source: Food Sci Nutr. 2018 Oct 10;6(8):2414–22. doi: 10.1002/fsn3.840 (PMC6261179; doi:10.1002/fsn3.840)
Supplement: Supplementary file 1 [file FSN3-6-2414-s001.doc]

Table. Retention time and contents of phenolic acid and flavanoid from RTL-EAF5 fraction

| Standard | Retention time (min) | EAF5-1  (µg/g ) | EAF5-2  (µg/g ) | EAF5-3  (µg/g ) | | EAF5-4  (µg/g ) | EAF5-5  (µg/g ) |
| --- | --- | --- | --- | --- | --- | --- | --- |
| Phenolic acid | | | | |  | | |
| *Syringic acid* | 23.66 | - | - | - | | 6.7 ± 0.9 | 27.3 ± 1.4 |
| *p-Coumaric acid* | 28.78 | - | - | - | | - | 95.0 ± 2.5 |
| Flavanoid | | | | |  | | |
| *Cirsimaritin* | 16.91 | - | - | - | | - | 805.5 ± 24.8 |

(-) Not detected.

Each value is means±SD (n=3).


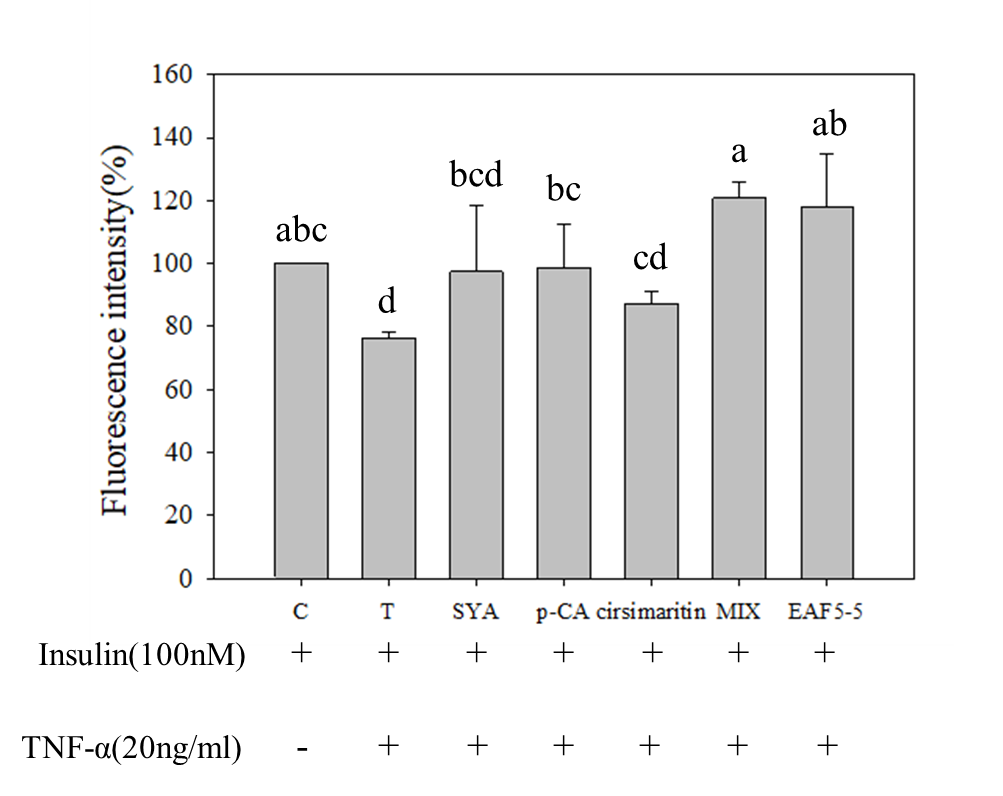


Figure. Effect of bioactive components in RTL on glucose uptake in mouse FL83B hepatocytes.

*significantly different from control. (*P* < 0.05)

Each value is means±SD (n=3).

C (Control)：FL83B cell incubated with F-12K medium.

T (TNF-α treated)：FL83B cell incubated with F-12K medium containing TNF-α (20ng/ml) for 16 hours to induced insulin resistance.

SYA：Syringic acid.

*p*-CA：*p*-coumaric acid.

MIX：Syringic acid+ *p*-coumaric acid+cirsimaritin.
